# In Vitro Evaluation of Iron-Induced Salivary Lipid Oxidation Associated with Exposure to Iron Nanoparticles: Application Possibilities and Limitations for Food and Exposure Sciences

**DOI:** 10.3390/ijerph17103622

**Published:** 2020-05-21

**Authors:** Susan Mirlohi

**Affiliations:** Department of Public Health, California State University Fresno, Fresno, CA 93740-8031, USA; susanmirlohi@csufresno.edu; Tel.: +1-559-278-7024

**Keywords:** zerovalent iron, oxidative stress, saliva, metallic flavor

## Abstract

Zerovalent iron nanotechnologies are widely used for groundwater remediation and increasingly considered for advance oxidation treatment in drinking water applications. Iron nanoparticles have been detected in drinking water systems and considered for food fortification; therefore, the potential for human exposure through ingestion can be a concern. This study aimed to assess whether ingestion of iron nanoparticles from drinking water could be detected through flavor perception using In Vitro salivary lipid oxidation as an indicator for metallic flavor perception. Ten female subjects, aged 29–59 years, donated saliva samples for use in the In Vitro experiments. Test samples consisted of 1:1 mixture of saliva and bottled drinking water (control) and three treatment solutions, spiked with ferrous sulfate, stabilized zerovalent iron nanoparticles (nZVI), and an aggregated/microsized suspension of mixed zerovalent iron and microsized suspension of iron and iron oxide metal powder, (mZVI). Upon mixing, samples were subjected to 15 min incubation at 37 °C to resemble oral conditions. Salivary lipid oxidation (SLO) was measured in all samples as micromoles of thiobarbituric acid reactive substances (TBARS)/mg Fe. Exposure to iron in all three forms induced significant amount of SLO in all treatment samples as compared to the control (*p* < 0.0001). The mean SLO levels were the highest in the ferrous treatment, followed by nZVI and mZVI treatments; the differences in the mean SLO levels were significant (*p* < 0.05). The findings indicate that oral exposure to stabilized ZVI nanoparticles may induce sensory properties different from that of ferrous salt, likely predictive of diminished detection of metallic flavor by humans.

## 1. Introduction

Metallic iron in its zerovalent form has been widely used in the treatment of contaminated groundwater since the early 1990s, when it was first discovered that chlorinated hydrocarbons, such as trichlorethene (TCE), could be dehalogenated in the presence of iron metal [1,2]. Since then, the use of metallic iron as applied in the form of permeable reactive barriers (PRBs) has been widely reported in groundwater remediation sites [3]. More recently, scientific explorations in the field of nanoscience have opened many more intriguing possibilities with reported uses of stabilized zerovalent iron nanoparticles (nZVI) for the removal of toxic contaminants, such as nitrate, perchlorates, arsenic, hexavalent chromium, uranium, and antibiotics, from soil and water matrices and the use of magnetic iron oxides for targeted drug delivery and food fortification [4,5,6,7,8,9,10,11]. Stabilized nZVI products consist of a thin layer of iron oxide to make nanoiron products more stable when exposed to air while maintaining reactivity for contaminant removal applications [12].

Environmental nanoparticles (NPs), sized 1 to 100 nm, are classified as naturally occurring or engineered. Naturally occurring NPs are ubiquitous in nature and exist in all environmental media: air, water, and the subsurface [13]. On the other hand, engineered NPs are intentionally produced for various applications in science, technology, medicine, industries, and daily consumer products [14]. Consequently, with widespread usage and increasing applications, concerns for environmental exposure and toxicity impacts arise. For nZVI, the most likely potential for human exposure is through accidental dermal contact during manufacturing and slurry application or ingestion of drinking water from contaminated wells and surface waters [15]. Inhalation can also be an important route of exposure through aerosols, iron oxide/carbon black particles, and atmospheric dust [16,17,18]. Few studies have investigated the toxicity impacts of nZVI in the aquatic environment due to the ubiquitous nature of iron in the form of oxides as well as the limited mobility of iron nanoparticles once oxidized to form ferrous, ferric, and iron oxides [19,20,21,22]. Although based on these characteristics, potential exposure to humans and higher organisms is believed to be unlikely, toxicity concerns are increasing as more engineered nanoparticles are being developed to enhance the mobility of nZVI through the use of particle stabilizing agents, such as surfactants, polymers, and polyelectrolytes. Additionally, the highly reductive property of nZVI that makes it so appealing in terms of removal of toxic contaminants has been recognized as a means by which surface and/or groundwater contaminants can be effectively captured and transported through drinking water systems. For example, nanoscale iron oxides have been found bounded to copper in surface waters many kilometers downstream from mining sites [18]. In a drinking water system, a sample of 20 nm particles was found to contain lead and iron; the iron was tentatively identified as being in its oxide form, hematite [13,18].

While elemental iron is a vital nutrient for the maintenance of body functions in all organisms, its toxicity potential is widely recognized due to its ability to cause oxidative stress in living cells by damaging membrane lipids and DNA. Oxidative stress is defined as an imbalance between production of free radicals and reactive oxygen species and their destruction by the protective actions of antioxidants and enzymes within the body [23]. Associated with inducing oxidative stress, excessive iron accumulation in the body has been linked to many serious diseases, such as atherosclerosis, Alzheimer’s disease, Parkinson’s disease, and cancer. Several types of metal nanoparticles, namely, titanium, silver, iron oxides, copper, manganese oxide, and aluminum, are known to induce inflammation in brain tissues and alter key protein functions that can ultimately lead to development of neurodegenerative diseases [24,25].

The ability of iron to induce oxidative stress in biological tissues and fluids has been quantified by measuring lipid oxidation products using the method of thiobarbituric acid reactive substances (TBARS). Lipid oxidation has been extensively studied using the TBARS assay, which involves derivatization of malondialdehyde (MDA) with thiobarbituric acid to produce a pink product that is quantified in a UV–VIS spectrophotometer [26]. Intriguingly, iron-induced salivary lipid oxidation in the oral cavity, as measured by TBARS, has been linked to the mechanism by which humans are able to detect the flavor of iron from the release of volatile and odorous by-products of lipid oxidation, such as MDA in the oral cavity [27]. 

The aims of this manuscript are to communicate findings of this study, which utilized salivary lipid oxidation (SLO) as an indirect measure of metallic flavor intensity to compare iron-induced SLO between soluble ferrous iron salt and nanoparticles of stabilized zerovalent iron products in micro- and nanoscale suspensions. The study aimed to explore whether the use of SLO can be a useful screening tool for predicting sensory properties and oxidative stress potentials from oral exposure to iron nanoparticles.

## 2. Materials and Methods

### 2.1. Human Subjects

This study was approved by the Institutional Review Board (IRB) at Virginia Tech (IRB Project No. 06-395). Human subjects were recruited from the community, students, faculty, and staff of Virginia Tech and Blacksburg, Virginia, by means of paper and email flyers. Subjects were required to have no chronic oral or general health problems, be nonsmokers, and not pregnant. All subjects read and signed an informed consent form in accordance with the approved IRB protocols. Ten female subjects, aged 29–59 years, donated saliva for use in this study.

### 2.2. Saliva Collection

Subjects were asked to refrain from eating, drinking, and smoking for at least one hour prior to collecting their saliva sample. Three daily saliva collection sessions were conducted within the same week and time frame of 10 a.m. to 12 noon. For each daily session, subjects were first asked to thoroughly rinse their mouth with Aquafina^®^ bottled water and wait 1 min to stabilize the conditions in their mouth. Then, subjects’ oral pH was measured using a pH indicator strip (Cen-Med/Fisher M95883; EMD Chemicals Inc.; Gibbstown, NJ, USA) by placing the pH strip on their tongue until it became moist with their saliva; then, using the color scale, the pH was read and recorded after 1 min and while the strip was still moist. Subjects were asked to expectorate approximately 4 mL of saliva into 50 mL propylene tubes during each session for a total volume of 12 mL collected over the three daily sessions. Between daily collections, saliva samples were kept stored in a freezer; at the end of final collection, saliva samples were frozen immediately and stored at −50 °C for up to one month until analysis. For long-term storage, temperature ranges of −20 to −80 °C have been shown to preserve stability of salivary constituent for up to 3 months [28].

### 2.3. Preparation of Iron Solutions

Three different stock solutions of iron were prepared immediately before use in the experiments. The stock solutions included iron (II) sulfate (Sigma-Aldrich, PA, CAS # 13463-43-g) and two aqueous dispersions of manufactured nZVI products, NANOFER 25S and NANOFER STAR (kindly provided by Nanoiron Ltd., Rajhrad, Czech Republic, EU). The iron solutions were prepared by mixing the iron salt or the nanoiron product with bottled drinking water (Aquafina^®^) to provide for 10 mg/L of total iron concentration in the resulting solution. For the NANOFER 25S, the suspension as provided by the manufacturer contained approximately 15% Fe(0), and it was diluted accordingly to the targeted concentration of 10 mg/L total Fe. The NANOFER STAR solution was prepared by mixing 1 part (10 g) of the powder with 4 parts (40 mL) of the bottled drinking water using a blender to provide for approximately 15% Fe(0) suspension; then, the mixture was diluted to the targeted concentration of 10 mg/L total Fe. As pH level is important in iron chemistry as well as stability of nanoparticles in suspension, pH was measured in the stock solutions of the prepared samples using pH indicator strips (Cen-Med/Fisher M95883). The total iron concentration of each stock solution was quantified using inductively coupled plasma mass spectroscopy (Thermo Electronic Corporation, X-Series ICP-MS, Waltham, MA, USA), following Standard Method 3120B from the American Public Health Association Standard Methods for Examination of Water and Wastewater [29].

### 2.4. Zerovalent Iron Nanoparticles Characteristics

Characteristics of the iron nanoparticle products were provided by the manufacturer (Nanoiron Ltd., Rajhrad, Czech Republic, EU). The NANOFER 25S is described as a stabilized water dispersion of nanoscale zerovalent iron consisting of Fe (14–18%), Fe_3_O_4_ (6–2%), carbon (0–1%), and a surfactant (3%) as an organic stabilizer. The NANOFER STAR is described as an air-stable highly reactive powder consisting of Fe(0) surface-stabilized nanoparticles coated by a thin organic surface layer to protect against air oxidation and allow for ease of transport and long-term storage. The powder mixture was composed of ≥80% iron and iron oxide. For both products, the average particle size was reported as <50 nm; the specific surface area was >25 m^2^/g with spherical morphology; the dispersion density was 1210 kg/cm^3^. As indicated by the product manufacturer, the NANOFER STAR is expected to behave like a micro-ZVI particle if the powder is applied directly to water without dispersing. To verify the particle size distribution and zeta potentials for freshly prepared suspensions, the iron nanoparticles were analyzed by dynamic light scattering (Zetasizer™ Nano Series; Beckman Coulter Inc., Brea, CA, USA). For reliable particle size and zeta potential determinations, the suspensions had to be diluted 100 times (i.e., approximately 0.15% Fe(0) solutions) and were filtered (1 µm) as necessary for particle size determination.

### 2.5. In Vitro Experiments

In Vitro experiments were performed using individual saliva samples from each of the 10 subjects as well as a single pooled saliva sample from multiple subjects. The pooled saliva sample consisted of a mixture of equal volumes of saliva from all 10 subjects. Saliva pooling was done to account for potential differences in experimental outcomes due to variation in salivary fluid between subjects. In Vitro test sample preparations followed procedures utilized in previous In Vivo (within the oral cavity) experiments for determination of ferrous iron sensory threshold and oral lipid oxidation measures in human subjects [30,31]. Thus, for this In Vitro study, test samples were prepared by mixing equal volumes of freshly thawed saliva in room temperature with freshly prepared iron stock or control solutions. The control sample consisted of equal volumes of saliva and bottled drinking water (Aquafina^®^). The choice of Aquafina^®^ as the control and dilution water was based on its low level of total dissolved solids (<10 mg/L) as used in a previous metallic flavor research study with human subjects [32]. The treatment groups consisted of equal volumes of saliva and iron stock solution specific to each treatment. The three treatment groups corresponding to each iron stock solution were identified as Fe(II) for saliva fluid reacted with ferrous iron; nZVI for saliva fluid reacted with iron nanoparticles using the NANOFER 25S product; and mZVI for saliva fluid reacted with microsized iron particles using the NANOFER STAR product. For each experimental run, a minimum of three replicates per control and treatment group were prepared. After preparing the mixture of saliva and test samples in 50 mL propylene test tubes, they were placed in a 37 °C water bath for 15 min to resemble oral cavity conditions. After the 15 min incubation time, test samples were immediately analyzed for salivary lipid oxidation using the TBARS method as a measure of iron-induced oxidative stress within the oral cavity. The TBARS method [33] was modified to work with liquid samples and to enhance readings at low concentrations [34]. Using the modified TBARS procedure, 1 mL of saliva samples and known concentrations of 1,1,3,3-tetramethoxypropane (MDA) standards were each mixed with 2 mL of prepared TBA solution consisting of 0.375% TBA, 0.506% sodium dodecyl sulfate (SDS), and 9.370% glacial acetic acid and digested for 60 min at 95 °C in a water bath. After digestions, samples were immediately cooled in an ice bath, mixed with 2 mL of n-butanol/pyridine mixture (at 15:1 ratio), and centrifuged for 15 min at a speed of 3000× *g*. The absorbance of the supernatant from each sample and standard was measured with a spectrophotometer at 532 nm. The concentration of TBARS was obtained from the standard curve and absorbance values. The standard curve was developed by running the TBARS method on known MDA standards at 0.03 to 10 μM concentrations. TBARS analysis was run in duplicates for each sample. As equal volumes of saliva and control or treatment solutions were mixed for In Vitro experiments, a 50% dilution factor was applied in the calculation to obtain the actual concentration of TBARS in each sample.

### 2.6. Data Analysis

Statistical software, JMP 9.0 (SAS, Cary, NC, USA), was used for all data analyses. One-way analysis of variance (ANOVA) and comparison of the means using Tukey’s honestly significant difference (HSD) or Wilcoxon/Kruskal–Wallis rank sum test were performed on the mean oxidative stress responses as measured by salivary lipid oxidation in the control and test samples. The SLO was also reported as delta SLO as the arithmetic difference between the measured salivary TBARS in the control and the iron-containing test samples. To normalize the measured iron-induced SLO levels, the delta SLO values in micromoles of TBARS were divided by the concentration of total iron in milligrams. All statistical analyses were performed at alpha level of 0.05, and results are presented as means ± standard error (SEM).

## 3. Results

### 3.1. Particle Size and Zeta Potential Characteristics of the Nanoparticles

**Figure 1:**
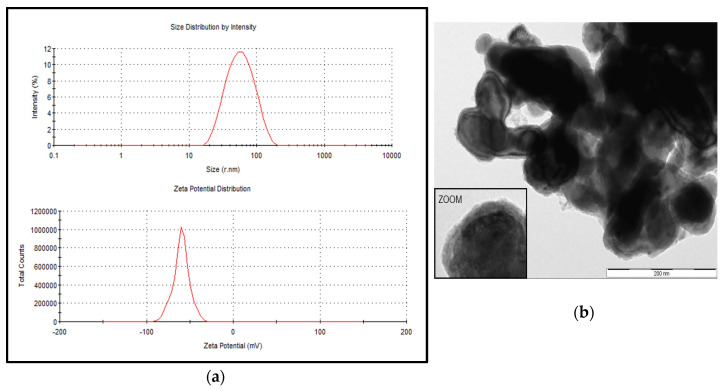
Particle size distribution and zeta potential for the stabilized suspension of zerovalent iron nanoparticles (nZVI). The nZVI products included NANOFER 25S (**a**) and NANOFER STAR (**b**). Both products were kindly provided by Nanoiron Ltd., Rajhrad, Czech Republic, EU.

The particle size distribution of the stabilized suspension of the iron nanoparticle product, NANOFER 25S, ranged from 18 to 110 nm with an average size of 52 nm; the zeta potential was measured at −60 mV (Figure 1a). The prepared suspension using the stabilized powder form, NANOFER STAR, behaved as micro—rather than nanoparticles, with the average size estimated at 635 nm and the zeta potential measured at −19 mV. Plots of size and zeta potential could not be obtained due to difficulty of obtaining a stable suspension; however, an electron microscopic view of the air-stabilized particles as provided by the manufacturer is shown in Figure 1b [35]. Based on this characterization, when the NANOFER 25S product was used in the In Vitro experiments, test samples were identified as nZVI, whereas designation of mZVI was used when the NANOFER STAR product was used, thus characterizing the particles as greater than 100 nm.

### 3.2. Measure of pH in Saliva and Test Samples

The pH levels in saliva ranged from 6.0 to 7.0 (mean = 6.4; SD = 0.33). The pH was 5.0 in the three iron stock solutions, while the measured pH was 7.0 in the control and all treatment samples.

### 3.3. Iron-Induced Oxidative Stress Response

**Figure 2:**
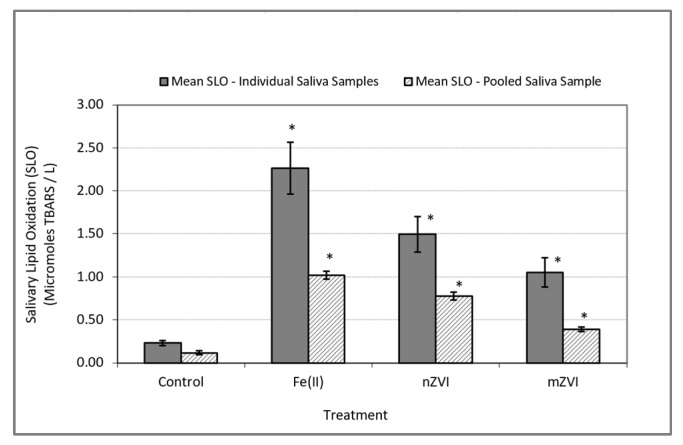
Oxidative stress response as measured by SLO using thiobarbituric acid reactive substances (TBARS). Solid bars represent mean SLO responses in In Vitro experiments using individual saliva samples from 10 subjects. Textured bars represent the mean SLO response in In Vitro experiments using a single pooled saliva sample for three duplicate experiments. Error bars constructed using one standard error from the mean. * indicate statistical significance (*p* < 0.05) in treatment group when compared to the control.

In the In Vitro experiments performed using individual saliva as well as the pooled saliva samples, the addition of iron to human saliva induced oxidative stress response. This was indicated by a significantly higher level of SLO in the test samples containing iron when compared to the control samples (Table 1). This observation was consistent in the experiments with individual saliva samples (*F* (3,45) = 15.60; *p* < 0.0001) as well as the pooled saliva sample (*F* (3,11) = 164.32; *p* < 0.0001) (Figure 2).

**Figure 3:**
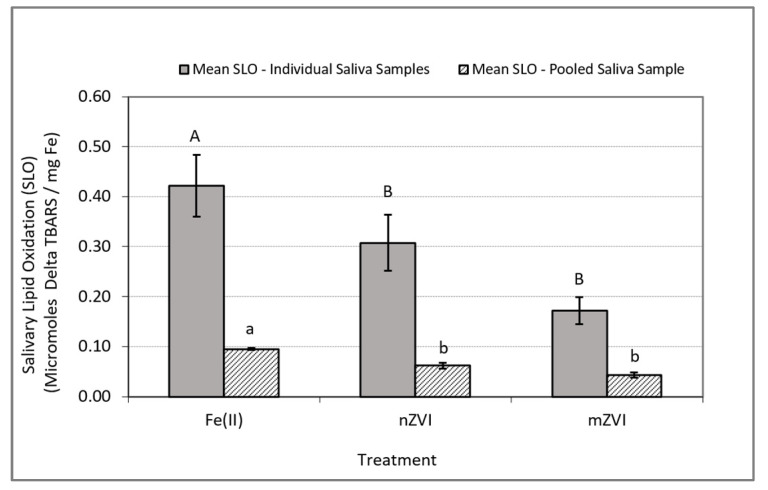
Iron-induced oxidative stress response as measured by delta SLO using TBARS. Delta SLO levels were normalized by milligram of total Fe. Blue bars represent mean SLO responses in In Vitro experiments using individual saliva samples from 10 to 13 subjects. Solid bars represent the mean SLO response in In Vitro experiments using a single pooled saliva sample for three replicate experiments using a single pooled saliva sample. Error bars constructed using one standard error from the mean. For each set of experiments, mismatched letters indicate statistical significance (*p* < 0.05) between the mean responses among the treatment groups.

In experiments conducted using individual saliva samples from the 10 to 13 human subjects, the analysis of variance on the iron-induced delta SLO levels (i.e., SLO response in treatment group minus SLO response in control group) indicated a significant difference between at least one pair of the mean responses (*F* (2, 35) = 3.72, *p* = 0.035). Follow-up comparison of the mean delta SLO responses using Wilcoxon’s test indicated a significance difference only between the Fe(II) and nZVI treatments (*p* = 0.038) and Fe(II) and mZVI (*p* = 0.044) but no significant differences between nZVI and mZVI (*p* = 0.47) (Table 1 and Figure 3).

In experiments conducted with the pooled saliva sample, the analysis of variance on the iron-induced delta SLO levels indicated a significant difference between at least one pair of the mean responses (*F* (2, 8) = 34.75, *p* = 0.0005). Follow-up comparison of the mean delta SLO responses using Tukey‘s HSD test indicated a significant difference between the treatment pairs Fe(II) and nZVI (*p* = 0.0045) and Fe(II) and mZVI (*p* = 0.0004) but no significant difference between nZVI and mZVI (*p* = 0.058) (Table 2 and Figure 3).

## 4. Discussion

### 4.1. Assessing the Impact of Nanoparticle Size and/or Aggregation on Iron-Induced Salivary Oxidative Stress Response

The findings from this study indicate that ferrous iron salt, being the most soluble compared to the two nanoiron products, induced the highest level of oxidative stress response in human salivary fluid as measured by lipid oxidation. After ferrous iron, the stabilized suspension of nZVI product, NANOFER 25S, produced the next highest level of iron-induced oxidative stress, with the stabilized powder, NANOFER STAR, showing the least oxidative stress response. Based on these observations, it is evident that, in the case of stabilized zerovalent iron composed of an outer layer of iron oxide, the potential for iron-induced salivary oxidative stress is higher when particle size becomes smaller, whereas the oxidative stress declines when particles get larger by size characteristics and/or the aggregation effect. Previous research studies exploring the impact of particle size on nanometals toxicity have produced varying results. In one study, nanometer or micrometer particle size toxicity varied by the type of metal; for example, copper oxide in its nanoform induced higher oxidative stress in human lung cells when compared to microsized particles. In the case of iron, iron oxide (Fe_2_O_3_) induced lower toxicity with no considerable difference between the particle sizes in terms of induced oxidative stress [36]. Other studies have shown that pH, particle surface chemistry, and presence of ligands are important factors in comparing toxicity levels of nZVI and ferrous iron [15,37,38].

### 4.2. Exploring the Role of pH and Zeta Potential on Iron Nanoparticles Behavior in Salivary Fluid

In aqueous systems, the role of pH in influencing zeta potential is critical as it indirectly influences particle charge and stability by changing the zeta potential in aqueous suspensions [39]. Nanosized particles can become attached to one another and effectively behave as microsized particles by physical aggregation [39,40]. Solution pH, as well as zeta potential, influences zerovalent nanoparticles’ tendencies to agglomerate [3,40]. Additionally, other factors, such as ionic strength and particle concentration, influence the agglomeration of particles [41]. Particles begin to agglomerate at zero zeta potential, thus becoming less mobile and reactive, while particles with large positive or negative zeta potentials are considered to be stable, thus resistant to agglomeration [42]. The particle stability range is defined at zeta potentials greater than +30 mV and less than −30 mV [42]. Previous research has indicated the zeta potential for uncoated nZVI to be −30 ± 3 mV, making them virtually immobile [43], while stabilized nZVI become increasingly mobile with higher zeta potentials, as high as −50 ± 1.2 mV [43]. In groundwater applications, nZVI have been shown to have 0 zeta potential at a pH of approximately 8.1. As the pH increases, zeta potential becomes negative, approaching −30 mV at pH 8.4; likewise, the zeta potential is positive below pH 8.1 [43].

In human saliva, the pH remains relatively neutral, with levels ranging from 5.5 to 7.5 [30,44]. The zeta potential of human saliva has been estimated to be 0 at pH of 3 and becomes negative with increasing pH levels, with a maximum zeta potential of −15 mV at pH 8.0 [45]. At the neutral pH of 7.0, a zeta potential of approximately −10 mV has been measured in human saliva [45]. Based on these saliva characteristics and with consideration of zeta potentials for nZVI, one can infer that with higher negative charge, stabilized nZVI may be less likely to agglomerate when combined with salivary fluid at pH 7.0 and thus be more reactive. On the other hand, already agglomerated nZVI with a smaller negative zeta potential may be more likely to reach 0 mV zeta potential once mixed with saliva. Additionally, interaction of surface-charged nZVI with the numerous salivary proteins present in human saliva can further impact their agglomeration properties [39,40]; this factor could in turn influence particle reactivity for inducing oxidative stress through interactions with lipids and proteins in saliva [46,47].

### 4.3. Predicting Flavor Perception of Iron Nanoparticles Based on SLO Phenomenon

In human sensory studies, the unpleasant flavor of iron in water has been described as “metallic” and “rusty nail”, leaving an aftertaste in the mouth of tasters [48]. Utilized as a chemical measure of metallic flavor intensity, iron-induced SLO in the oral cavity has been associated with the perception of metallic flavor as described by human subjects [30,49] and has been linked with retronasal detection of off-flavor odors and “fishy” aftertaste produced from volatile by-products of lipid oxidation in the oral cavity, namely, odorous aldehydes and ketones [27,50]. Previous research has demonstrated that the oral intake of iron-spiked drinking water results in a significant increase in In Vivo salivary lipid oxidation as measured by TBARS concentration in saliva before and after the oral intake of iron [30,49]. These findings are consistent with this In Vitro study on iron-induced SLO. Regarding metallic flavor perception, the significantly reduced iron-induced salivary lipid oxidation by stabilized water dispersion of nZVI and mZVI observed in this study can be predictive of diminished taste and flavor properties of iron nanoparticles compared to that of ferrous salt. Reduced SLO may correspond to less metallic flavor detection by humans, although reduced smell functions, which are important in retronasal detection of metallic flavor, can also play a role as nose closure has been associated with significant decline or loss of metallic flavor perception by humans [49,51,52]. With application in food fortification, previous research has indicated that nanoscale iron particles used as food additives impart considerably less color and taste change in food matrices. Additionally, being less soluble than ferrous sulfate, iron nanoparticles were demonstrated to be highly bioavailable when fed to rats in iron-fortified food matrices [6]. In terms of advantages and drawbacks, a higher bioavailability, without diminishing impacts on sensory properties, would translate to benefits for iron-deficient populations [10,53] and potential for overexposure risk to vulnerable populations with hereditary iron overload disease [54].

### 4.4. Potential Application of In Vitro SLO For Comparing Toxicity Of Metals and Their Associated Nanoparticles in Biological Fluids

Exposure of humans to iron and other essential, trace, or toxic metals and their associated nanoparticles are likely scenarios as they are widely present in the environment through natural and engineered systems and products [13,55]. Therefore, assessing the potential of metal toxicity to humans and other living organisms remains a continuing need and concern. As utilized by this research on iron-induced SLO of ferrous sulfate and stabilized nZVI, metal-induced salivary lipid oxidation through measurement of TBARS can offer a simple In Vitro screening approach for assessing relative reactivity and oxidative stress potentials of iron and iron oxide nanoparticles as well as other metals and metallic nanoparticles of toxicity concern. The measurement of TBARS has been a widely used method for screening and monitoring lipid peroxidation since the early 1980s to evaluate samples that include human and animal tissues and fluids, drugs, and foods [26]. More recently, with developments in methods involving analysis by GC–MS and LC–MS, the use of the TBARS method is not regarded as specific enough for use in complex matrices, such as human plasma, as it does not directly measure MDA as a common by-product of lipid oxidation and a measure of oxidative stress [56]. However, the TBARS method can be a useful screening method in circumstances where access to sensitive analytical instrumentation for detection and measurement of specific by-products of lipid oxidation is limited. Screening for oxidative stress through measurement of In Vitro salivary lipid oxidation could help in comparing the toxicity of metals when exposed to humans through intake of water and other beverages. Additionally, as salivary fluid contains proteins as well as lipids, measure of SLO could be supplemented by oxidative stress measurements of other biomarkers of lipid and protein oxidation, such as F2-isoprostanes, protein carbonyls, 4-hydroxy-2-nonenal (HNE), and specific aldehyde compounds [57,58,59,60,61].

## 5. Conclusions

With increasing and innovative use of iron nanoparticles in a variety of applications, concerns for potentially toxic exposure to humans as well as other organisms are well founded. Equally important are considerations for the positive benefits of using iron nanoparticles for enhancing food quality and nutrition [6,10]. This is an important application benefit as iron deficiency is a global problem affecting nearly 2 million people worldwide, mostly in developing countries and among children and pregnant women [62,63]. Additionally, as human senses are often the first line of defense in detecting contaminants as well as choosing to consume aesthetically pleasing food and beverage products, the results of this study are relevant for predicting the ability of humans to detect stabilized iron nanoparticles through oral exposure. Reduced level of lipid oxidation is an indication of lower toxicity while also being an indicator of reduced sensory response and potential for iron ingestion above safe levels of exposure.

For improved detection ability, In Vitro lipid oxidation studies using human saliva as a biomarker of exposure to metals should be conducted with pooled saliva in order to reduce variability among human subjects. As indicated by this research, coefficient of variation for SLO response could range from 45% to 60% when using individual saliva samples, whereas it was reduced to 3% to 16% with the pooled saliva sample, thus enhancing the ability to detect significant differences between the mean SLO responses among different treatment groups for exposure studies. Reducing variability is especially important for using the test as a screening method.

Lastly, with human salivary fluid enriched with numerous proteins, including those with metal-binding capacities, fatty acids and lipids, electrolytes, and antimicrobial agents, human saliva, in real or simulated formulation, can be more widely used as an innovative and simple means by which interactions and toxicity effects of stabilized nZVI and other metal nanoparticles in biological fluids can be studied and compared.

## Figures and Tables

**Table 1:** Summary of oxidative stress response as measured by In Vitro salivary lipid oxidation (SLO) in experiments with individual saliva samples (*n* = 10 samples for control and Fe(II) treatments; *n* = 13 samples for nZVI and mZVI* treatments).

Treatment	Mean SLO (μM TBARS) *	SEM ^2^ [95% Confidence Interval]	Delta SLO ^3^ (μM TBARS/mg Fe)	SEM [95% Confidence Interval]
Control	0.229 (SD ^1^ = 0.098)	0.031 [0.159–0.299]	-	-
Fe(II)	2.26 (SD ^1^ = 0.959)	0.303 [1.578–2.95]	0.421 (SD ^1^ = 0.195)	0.062 [0.282–0.561]
nZVI	1.49 (SD ^1^ = 0.751)	0.208 [1.023–1.97]	0.307 (SD ^1^ = 0.202)	0.056 [0.181–0.434]
mZVI	1.05 (SD ^1^ = 0.620)	0.172 [0.662–1.44]	0.172 (SD ^1^ = 0.096)	0.027 [0.112–0.232]

^1^ SD: standard deviation; ^2^ SEM: standard error of the mean; ^3^ delta SLO: SLO in the treatment sample minus the SLO in the control sample. SLO levels were normalized based on the measured amount of total iron in the sample. * Definition of terms: TBARS, thiobarbituric acid reactive substances. mZVI: microsized suspension of iron and iron oxide particles using the NANOFER STAR product nZVI: stabilized zerovalent iron nanoparticles.

**Table 2:** Summary of oxidative stress response as measured by In Vitro SLO in experiments with pooled saliva samples (*n* = 3 replicate samples of one pooled saliva sample from all subjects was used for control and each treatment).

Treatment	Mean SLO (μM TBARS)	SEM ^2^ [95% Confidence Interval]	Delta SLO ^3^ (μM TBARS/mg Fe)	SEM [95% Confidence Interval]
Control	0.117 (SD ^1^ = 0.041)	0.023 [0.016–0.218]	-	-
Fe(II)	1.020 (SD = 0.077)	0.045 [0.828–1.212]	0.095 (SD = 0.003)	0.002 [0.092–0.098]
nZVI	0.777 (SD = 0.080)	0.046 [0.577–0.977]	0.062 (SD = 0.010)	0.006 [0.052–0.072]
mZVI	0.389 (SD = 0.044)	0.025 [0.280–0.498]	0.043 (SD = 0.008)	0.005 [0.035–0.052]

^1^ SD: standard deviation; ^2^ SEM: standard error of the mean; ^3^ delta SLO: SLO in the treatment sample minus the SLO in the control sample. SLO levels were normalized based on the measured amount of total iron in the sample.
